# Supplementary material for: Oncolytic virotherapy induced CSDE1 neo-antigenesis restricts VSV replication but can be targeted by immunotherapy
Source: Nat Commun. 2021 Mar 26;12:1930. doi: 10.1038/s41467-021-22115-1 (PMC7997928; doi:10.1038/s41467-021-22115-1)
Supplement: Supplementary file 1 — Supplementary Information [file 41467_2021_22115_MOESM1_ESM.pdf]

SUPPLEMENTARY INFORMATION

Oncolytic Virotherapy Induced CSDE1 Neo-antigenesis Restricts VSV Replication But Can Be Targeted By Immunotherapy

Supplementary Figure 1

Sanger sequencing shown begining at position 7-14 in CSDE1

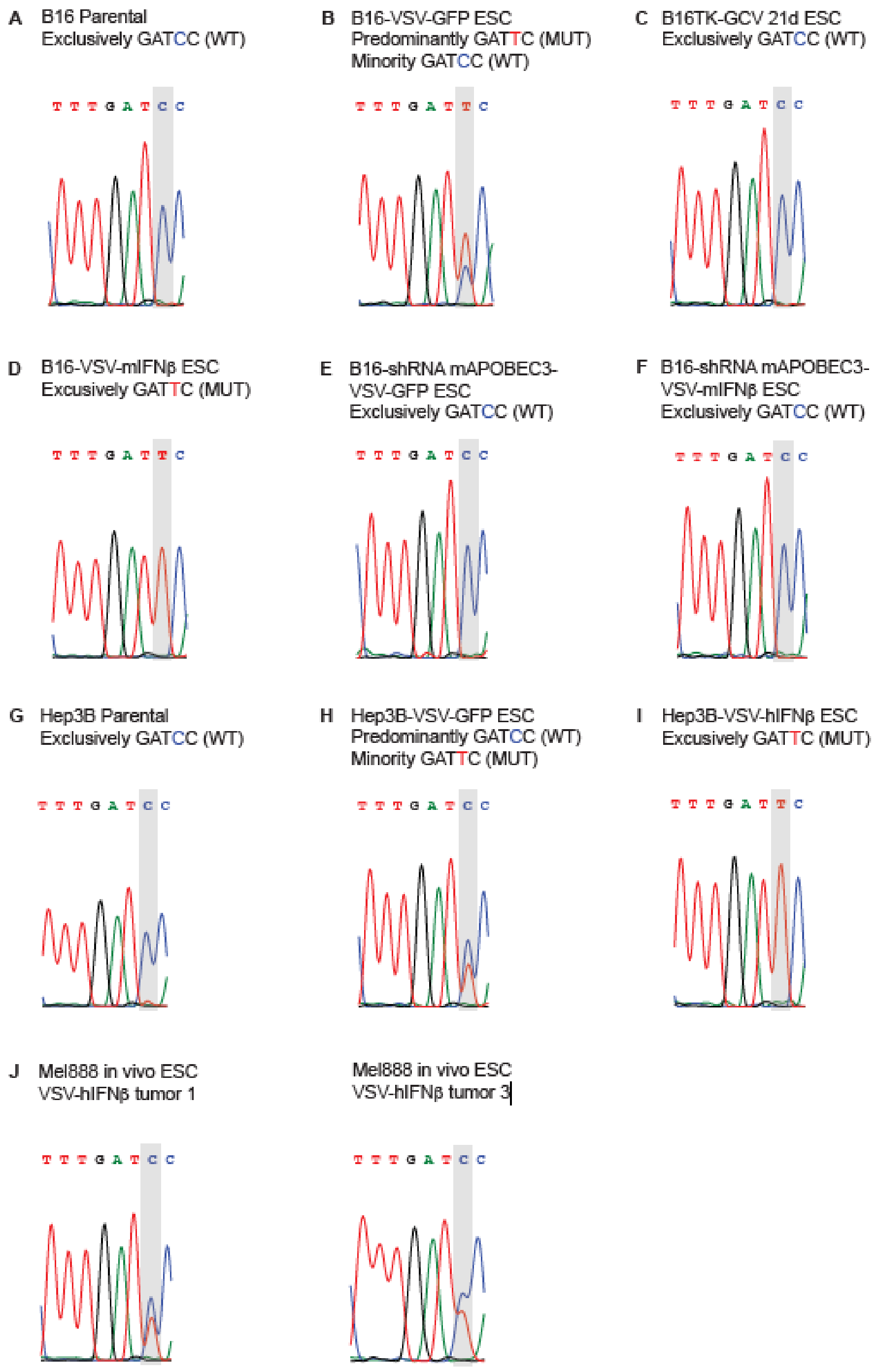

Supplementary Figure 1. Escape from VSV-IFN-ß selects for the CSDE1^{C-T} mutation. B16 murine melanoma or Hep3B human HCC cells were infected (MOI 0.01) for 21d as in **Methods** and [1]. Surviving cells were pooled and genomic DNA prepared. Sanger sequencing of CSDE1 is shown for: **A.** Parental, uninfected B16 showing a homogenous population of wild type CSDE1 sequence; **B.** B16-VSV-GFP-ESC showing a mixed population of escape cells with either wild type ATCC or mutated ATTC sequence; **C.** B16 cells stably expressing the HSVtk suicide gene [2], selected in GCV for 21d; **D.** B16-VSV-IFNß-ESC showing a nearly homogenous population of escape cells with mutated ATTC sequence; **E&F.** B16 cells stably expressing shRNA against APOBEC3 as in [1] selected for escape from VSV-GFP (**E**) or VSV-IFNß (**F**); **G.** Parental, uninfected Hep3B; **H.** Hep3B-VSV-GFP-ESC; and **I.** Hep3B-VSV-IFNß-ESC. **J.** Nude mice bearing s.c. Mel888 human melanoma tumors treated with i.t. VSV-hIFNß as described in [1] were excised upon escape from treatment, genomic DNA prepared and Sanger sequencing used to characterize the CSDE1 gene as shown. 4 of 4 excised, VSV-hIFNß-escaped tumors had mixed populations of CSDE1^{WT} and CSDE1^{C-T} tumor cells at ratios close to 1:1. Results are representative of seven separate clones (**A-D**); 3 separate clones (**E,F**) and (**G-I**); and 4 separate escape tumors (**J**).

Supplementary Figure 2

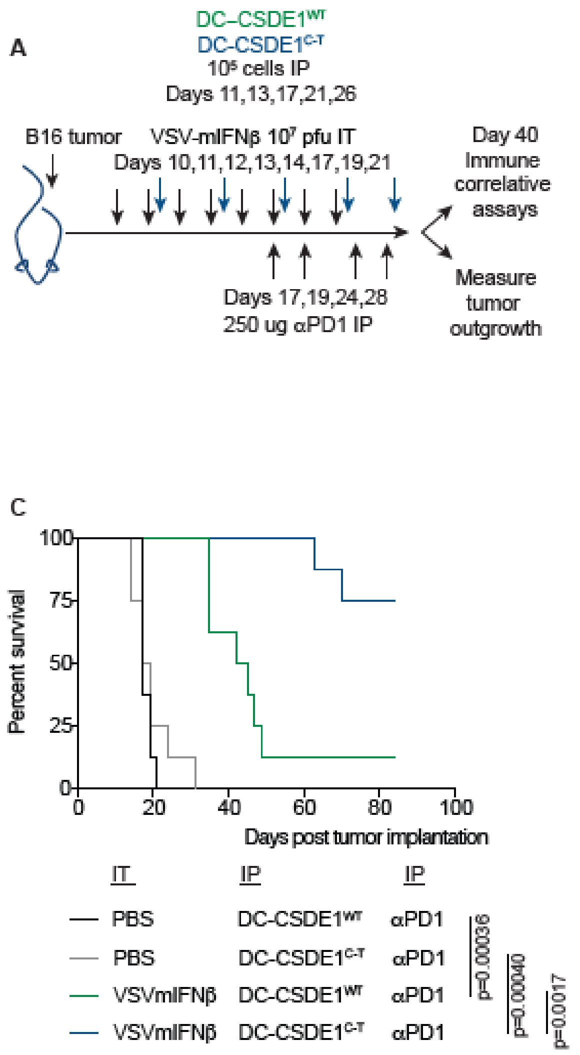

Supplementary Figure 2. DC-CSDE1$^{C\text{-}T}$ vaccination. A. *In vitro* IL-4/GM-CSF matured murine DC were transfected with nothing, CSDE1^{WT} or CSDE1$^{C\text{-}T}$ expression plasmids and 48 hrs later administered as vaccines with i.t. treatments with VSV-mIFN-ß and α-PD1. **B.** On d40, spleens and LN were assayed for IFN-γ following *in vitro* re-stimulation with live B16 cells or with live B16 cells stably expressing CSDE1$^{C\text{-}T}$. WT: DC vaccines expressing wild type CSDE1; * DC vaccine expressing mutated CSDE1$^{C\text{-}T}$. **C.** C57Bl/6 mice with 10d B16 tumors were injected i.t. with PBS or with VSV-mIFNß, (10^7pfu/injection) and i.p. with unloaded DC (DC), DC vaccine expressing wild type CSDE1 (DC-CSDE1^{WT}) or with DC vaccine expressing mutated CSDE1$^{C\text{-}T}$ (CSDE1$^{C\text{-}T}$) followed by α-PD-1 (n=8/grp) as shown in **A**. Kaplan-Meier survival for groups is shown. Representative of two separate experiments. P-values were determined using the Log-rank Mantel Cox test. For multiple comparisons using the Bonferroni correction, overall statistical significance threshold was set at $\alpha = 0.05$ (3 comparisons at $p<0.0167$). **D.** Mice were injected as in **A.** (3 mice/grp). 24hrs after the last injection of virus (d22), or earlier if tumor size excised 1.0cm diameter in the PBS groups, tumors were excised and assayed for IL-12 or TNF-α by ELISA (normalized by protein concentration in whole tumor cell lysates and expressed as pg/ml protein). Representative of two separate experiments. Each symbol in B,D represents a mouse (n=2 or 3/group). Means $\pm$ SD are shown. ND, not detected (below limit of detection). P-values were determined using a two-way ANOVA (B) with a Tukey multiple comparisons post-test. Statistical significance set at $p < 0.05$ for B. Source data are provided as a Source Data file.

Supplementary Figure 3

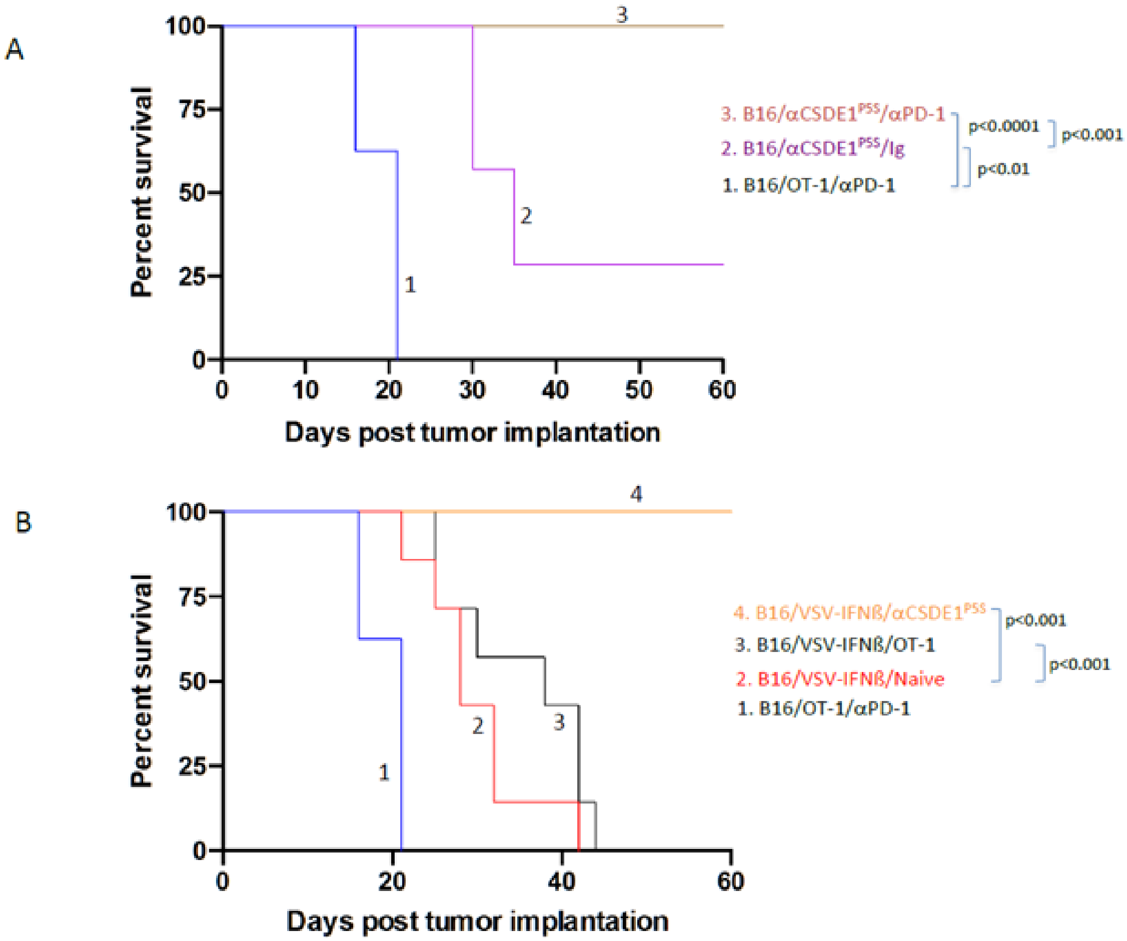

Supplementary Figure 3: C57Bl/6 mice with 10d established s.c. B16 tumors (7 mice/grp) were treated **A.** i.v. with $2.5x10^6$ OT-1 T cells activated *in vitro* for 5d with IL-2 (50 IU rhIL-2/ ml) and SIINFEKL peptide (1µg/ml) (d10), and with anti-PD-1 antibody i.p. (300µg/injection) (d17,19,21); or with $2.5x10^6$ CD8+ T cells recovered from the spleens of mice which had rejected B16 tumors treated with VSV-CSDE1^{P5S} (**Fig.5F**) and activated *in vitro* for 5d with IL-2 and the CSDE1^{P5S} mutated peptide MSFDSNLLH (1µg/ml) (d10) either with, or without, anti-PD-1 antibody (d17,19,21). **B.** 3 additional groups were treated intratumorally with VSV-IFN-ß (d10,12,14) ($5x10^7$ pfu/injection) and subsequently with either CD8+ T cells from naïve C57Bl/6 mice (d17); *in vitro* activated OT-I CD8+ T cells; or *in vitro* activated anti-CSDE1^{P5S} CD8+ T cells. Survival with time is shown. Data (A, B) from a single experiment not repeated. P-values were determined using the Log-rank Mantel Cox test. Source data are provided as a Source Data file.

Supplementary References

1 Huff, A. L. *et al.* APOBEC3 Mediates Resistance to Oncolytic Viral Therapy. *Mol Ther Oncolytics* **11**, 1-13, doi:10.1016/j.omto.2018.08.003 (2018).

2 Evgin, L. *et al.* Suboptimal T-cell Therapy Drives a Tumor Cell Mutator Phenotype That Promotes Escape from First-Line Treatment. *Cancer Immunol Res* **7**, 828-840, doi:10.1158/2326-6066.CIR-18-0013 (2019).
