# Supplementary material for: Oncolytic virotherapy induced CSDE1 neo-antigenesis restricts VSV replication but can be targeted by immunotherapy
Source: Nat Commun. 2021 Mar 26;12:1930. doi: 10.1038/s41467-021-22115-1 (PMC7997928; doi:10.1038/s41467-021-22115-1)
Supplement: Supplementary file 3 — Reporting Summary [file 41467_2021_22115_MOESM3_ESM.pdf]

nature research

Corresponding author(s): Richard Vile

Last updated by author(s): Feb 18, 2021

Reporting Summary

Nature Research wishes to improve the reproducibility of the work that we publish. This form provides structure for consistency and transparency in reporting. For further information on Nature Research policies, see our Editorial Policies and the Editorial Policy Checklist.

Statistics

For all statistical analyses, confirm that the following items are present in the figure legend, table legend, main text, or Methods section.

n/a	Confirmed	
[ ]	[x]	The exact sample size (*n*) for each experimental group/condition, given as a discrete number and unit of measurement
[x]	[ ]	A statement on whether measurements were taken from distinct samples or whether the same sample was measured repeatedly
[ ]	[x]	The statistical test(s) used AND whether they are one- or two-sided<br>*Only common tests should be described solely by name; describe more complex techniques in the Methods section.*
[ ]	[x]	A description of all covariates tested
[ ]	[x]	A description of any assumptions or corrections, such as tests of normality and adjustment for multiple comparisons
[ ]	[x]	A full description of the statistical parameters including central tendency (e.g. means) or other basic estimates (e.g. regression coefficient) AND variation (e.g. standard deviation) or associated estimates of uncertainty (e.g. confidence intervals)
[ ]	[x]	For null hypothesis testing, the test statistic (e.g. *F*, *t*, *r*) with confidence intervals, effect sizes, degrees of freedom and *P* value noted<br>*Give P values as exact values whenever suitable.*
[x]	[ ]	For Bayesian analysis, information on the choice of priors and Markov chain Monte Carlo settings
[x]	[ ]	For hierarchical and complex designs, identification of the appropriate level for tests and full reporting of outcomes
[x]	[ ]	Estimates of effect sizes (e.g. Cohen's *d*, Pearson's *r*), indicating how they were calculated

Our web collection on statistics for biologists contains articles on many of the points above.

Software and code

Policy information about availability of computer code

Data collection	Predicted MHC binding affinities were performed using the NETMHC 4.0 server. (http://www.cbs.dtu.dk/services/NetMHC/).
Data analysis	Data analysis was done using Graphpad prism version 8 and GraphPad QuickCalcs online tool (https://www.graphpad.com/quickcalcs/randMenu/)

For manuscripts utilizing custom algorithms or software that are central to the research but not yet described in published literature, software must be made available to editors and reviewers. We strongly encourage code deposition in a community repository (e.g. GitHub). See the Nature Research guidelines for submitting code & software for further information.

Data

Policy information about availability of data

All manuscripts must include a data availability statement. This statement should provide the following information, where applicable:

- Accession codes, unique identifiers, or web links for publicly available datasets
- A list of figures that have associated raw data
- A description of any restrictions on data availability

All data associated with this study are present in this paper or in the accompanying Supplementary Materials.

Field-specific reporting

Life sciences study design

All studies must disclose on these points even when the disclosure is negative.

Sample size	The number of seven animals per group was determined as the minimal number of animals required per group in order to make statistically relevant comparisons between groups while accounting for experimental error. When working with animal models, there is a significant amount of variability that needs to be taken into consideration so experiment repetition gives us confidence that such group and/or batch-to-batch differences. This number was calculated with consultation from a Mayo statistician using alpha=0.05 level, two sided log-rank test for equality of survival curves will have approximately 80% power to detect a difference between a control group and a treatment group. For in vitro studies, a minimum of triplicates was used to allow for the calculation of statistics.
Data exclusions	No data was excluded.
Replication	All of the in vivo experiments described in the main text our manuscript were repeated at least once and, in most cases, multiple times with similar results. Specifically, 5f repeated twice, S2c repeated once, S3A and B were run only once. Representative data shown as noted in the manuscript. For other experiments, number of repeats are indicated in the figure legend.
Randomization	For in vivo studies, mice were randomized at time of tumor implantation using the GraphPad QuickCalcs online tool (https://www.graphpad.com/quickcalcs/randMenu/). For in vitro studies, no randomization was performed as cell used in this study were pulled from a single preparation with no reason to believe that the spacial location in the well impacted results.
Blinding	Tumors were measured by a single blinded individual. For in vitro studies The investigators were not blinded to the allocation of groups during experiments or subsequently during the analysis. Fully blinded<br>experiments were not possible due to personnel availability to accommodate such situations.

Reporting for specific materials, systems and methods

We require information from authors about some types of materials, experimental systems and methods used in many studies. Here, indicate whether each material, system or method listed is relevant to your study. If you are not sure if a list item applies to your research, read the appropriate section before selecting a response.

Materials & experimental systems

n/a	Involved in the study	
☐	☒	Antibodies
☐	☒	Eukaryotic cell lines
☒	☐	Palaeontology and archaeology
☐	☒	Animals and other organisms
☐	☒	Human research participants
☒	☐	Clinical data
☒	☐	Dual use research of concern

Methods

n/a	Involved in the study	
☒	☐	ChIP-seq
☒	☐	Flow cytometry
☒	☐	MRI-based neuroimaging

Antibodies

Antibodies used	Anti-CSDE1 (Polyclonal, Bethyl Laboratories Cat# A303-160A), Anti-PD1 (RMP1-14, BioXCell Cat# BE0146), Rat IgG (Isotype Control, Jackson ImmunoResearch Cat# 012-000-003), Anti-VSV M (Clone 23H12, EMD Millipore Corp Cat# MABF2347 Lot Q3418256), Anti-Rabbit Secondary (ECL IgG HRP, Sigma Aldrich GENA935), Anti-Mouse Secondary (ECL IgG HRP, Sigma Aldrich Cat# NA931V)
Validation	All antibodies used were validated by the manufacturer.<br><br>Anti-CSDE1was validated using Hela, 293T, and NIH3T3 cell lysates<br>https://www.bethyl.com/product/pdf/A303-160A.pdf<br><br>Anti-PD1 was validated on purified mouse PD1<br>https://bxcell.com/product/invivomab-anti-m-pd-1/<br><br>Rat IgG was validated based on immunoelectrophoresis paterns<br>https://www.jacksonimmuno.com/catalog/products/012-000-003<br><br>Anti-VSV M was validated by western blot<br>https://www.emdmillipore.com/US/en/product/Anti-VSV-Matrix-Protein-M-Antibody-clone-23H12,MM_NF-MABF2347-100UG#overview<br><br>Anti-rabbit secondary was validated by immunoaffinity chromatography<br>https://www.sigmaaldrich.com/catalog/product/sigma/sab3700934?lang=en&region=US

Anti-mouse secondary was validated by western blot<br>https://www.sigmaaldrich.com/catalog/product/sigma/gena9311ml?lang=en&region=US

Eukaryotic cell lines

Policy information about cell lines

Cell line source(s)	B16.F1 murine melanoma, Hep3B human hepatocellular carcinoma, and BHK hamster kidney cells were obtained from the ATCC. B16TK cells were derived from a B16.F1 clone transfected with a plasmid expressing the Herpes Simplex Virus thymidine kinase (HSV-1 TK) gene in 1997/1998. Mel888 cells were obtained from the Imperial Cancer Research Fund (ICRF) in 1997/1998. CSDE1-WT and CSDE1-C-T over expression cell lines were generated by transfection of pcDNA3.1 expression vectors (B16, Hep3B, and Mel888).
Authentication	B16.F1 cells have been authenticated using markers specific to B16.F1 cells. The other cell lines have not been authenticated.
Mycoplasma contamination	All cell lines are tested for mycoplasma quarterly and are shown to be negative.
Commonly misidentified lines<br>(See ICLAC register)	No commonly misidentified cell lines were used in this study.

Animals and other organisms

Policy information about studies involving animals; ARRIVE guidelines recommended for reporting animal research

Laboratory animals	6-8 week old female C57BL/6 mice were purchased from Jackson Laboratories (Bar Harbor, Maine).<br>4-6 week old female athymic nude mice (Foxn1nu/nu) were purchased from Envigo (Indianapolis, Indiana)
Wild animals	This study did not involve wild animals.
Field-collected samples	This study did not involve collection of samples in the field.
Ethics oversight	All in vivo studies were approved by the Institutional Animal Care and Use Committee at Mayo Clinic.

Note that full information on the approval of the study protocol must also be provided in the manuscript.

Human research participants

Policy information about studies involving human research participants

Population characteristics	PBMCs were obtained from apheresis cones from healthy donors. For donors 1-5: 3 males and 2 females aged 33-56. The remaining donors are unknown.
Recruitment	Participants were not recruited specifically for this study. Samples used were obtained as a byproduct of healthy volunteers donating plasma.
Ethics oversight	Informed consent was obtained from all donors for the use of their sample for research purposes. Authorization to use the samples was approved by the Division of Transfusion Medicine Research Committee at Mayo Clinic. Samples were determined to be IRB exempt.

Note that full information on the approval of the study protocol must also be provided in the manuscript.
